# A haplotype-resolved genome assembly of seed hemp (*Cannabis sativa*) and analysis of Y chromosome divergence from the X

**DOI:** 10.1093/hr/uhaf268

**Published:** 2025-09-28

**Authors:** Huawei Wei, Zuqing Yang, Lingling Zhuang, Xueqing Pan, Haifeng Jia, Shaolian Jiang, Qin Li, Jiantang Xu, Aifen Tao, Pingping Fang, Jianmin Qi, Ray Ming, Liwu Zhang

**Affiliations:** Key Laboratory of Ministry of Education for Genetics, Breeding and Multiple Utilization of Crops, Fujian Agriculture and Forestry University, Fuzhou 350002, China; Fujian Public Platform for Germplasm Resources of Bast Fiber Crops/Fujian Provincial Key Laboratory of Crop Breeding by Design, Fujian Agriculture and Forestry University, Fuzhou 350002, China; Center for Genomics and Biotechnology, Haixia Institute of Science and Technology, Fujian Agriculture and Forestry University, Fuzhou 350002, China; Bozhou Key Laboratory of Biosynthesis of Effective Components of Medicinal Plants/Anhui Engineering Research Center for Development and Application of Functional Blended Liquor, Bozhou University, Bozhou 236800, China; Key Laboratory of Ministry of Education for Genetics, Breeding and Multiple Utilization of Crops, Fujian Agriculture and Forestry University, Fuzhou 350002, China; Fujian Public Platform for Germplasm Resources of Bast Fiber Crops/Fujian Provincial Key Laboratory of Crop Breeding by Design, Fujian Agriculture and Forestry University, Fuzhou 350002, China; Center for Genomics and Biotechnology, Haixia Institute of Science and Technology, Fujian Agriculture and Forestry University, Fuzhou 350002, China; Key Laboratory of Ministry of Education for Genetics, Breeding and Multiple Utilization of Crops, Fujian Agriculture and Forestry University, Fuzhou 350002, China; Fujian Public Platform for Germplasm Resources of Bast Fiber Crops/Fujian Provincial Key Laboratory of Crop Breeding by Design, Fujian Agriculture and Forestry University, Fuzhou 350002, China; Center for Genomics and Biotechnology, Haixia Institute of Science and Technology, Fujian Agriculture and Forestry University, Fuzhou 350002, China; Key Laboratory of Ministry of Education for Genetics, Breeding and Multiple Utilization of Crops, Fujian Agriculture and Forestry University, Fuzhou 350002, China; Fujian Public Platform for Germplasm Resources of Bast Fiber Crops/Fujian Provincial Key Laboratory of Crop Breeding by Design, Fujian Agriculture and Forestry University, Fuzhou 350002, China; Center for Genomics and Biotechnology, Haixia Institute of Science and Technology, Fujian Agriculture and Forestry University, Fuzhou 350002, China; Center for Genomics and Biotechnology, Haixia Institute of Science and Technology, Fujian Agriculture and Forestry University, Fuzhou 350002, China; Key Laboratory of Ministry of Education for Genetics, Breeding and Multiple Utilization of Crops, Fujian Agriculture and Forestry University, Fuzhou 350002, China; Fujian Public Platform for Germplasm Resources of Bast Fiber Crops/Fujian Provincial Key Laboratory of Crop Breeding by Design, Fujian Agriculture and Forestry University, Fuzhou 350002, China; Center for Genomics and Biotechnology, Haixia Institute of Science and Technology, Fujian Agriculture and Forestry University, Fuzhou 350002, China; Key Laboratory of Ministry of Education for Genetics, Breeding and Multiple Utilization of Crops, Fujian Agriculture and Forestry University, Fuzhou 350002, China; Fujian Public Platform for Germplasm Resources of Bast Fiber Crops/Fujian Provincial Key Laboratory of Crop Breeding by Design, Fujian Agriculture and Forestry University, Fuzhou 350002, China; Center for Genomics and Biotechnology, Haixia Institute of Science and Technology, Fujian Agriculture and Forestry University, Fuzhou 350002, China; Key Laboratory of Ministry of Education for Genetics, Breeding and Multiple Utilization of Crops, Fujian Agriculture and Forestry University, Fuzhou 350002, China; Fujian Public Platform for Germplasm Resources of Bast Fiber Crops/Fujian Provincial Key Laboratory of Crop Breeding by Design, Fujian Agriculture and Forestry University, Fuzhou 350002, China; Key Laboratory of Ministry of Education for Genetics, Breeding and Multiple Utilization of Crops, Fujian Agriculture and Forestry University, Fuzhou 350002, China; Fujian Public Platform for Germplasm Resources of Bast Fiber Crops/Fujian Provincial Key Laboratory of Crop Breeding by Design, Fujian Agriculture and Forestry University, Fuzhou 350002, China; Key Laboratory of Ministry of Education for Genetics, Breeding and Multiple Utilization of Crops, Fujian Agriculture and Forestry University, Fuzhou 350002, China; Fujian Public Platform for Germplasm Resources of Bast Fiber Crops/Fujian Provincial Key Laboratory of Crop Breeding by Design, Fujian Agriculture and Forestry University, Fuzhou 350002, China; Key Laboratory of Ministry of Education for Genetics, Breeding and Multiple Utilization of Crops, Fujian Agriculture and Forestry University, Fuzhou 350002, China; Fujian Public Platform for Germplasm Resources of Bast Fiber Crops/Fujian Provincial Key Laboratory of Crop Breeding by Design, Fujian Agriculture and Forestry University, Fuzhou 350002, China; Center for Genomics and Biotechnology, Haixia Institute of Science and Technology, Fujian Agriculture and Forestry University, Fuzhou 350002, China; Department of Plant Biology, The University of Illinois at Urbana-Champaign, Urbana, IL 61801, USA; Key Laboratory of Ministry of Education for Genetics, Breeding and Multiple Utilization of Crops, Fujian Agriculture and Forestry University, Fuzhou 350002, China; Fujian Public Platform for Germplasm Resources of Bast Fiber Crops/Fujian Provincial Key Laboratory of Crop Breeding by Design, Fujian Agriculture and Forestry University, Fuzhou 350002, China; Center for Genomics and Biotechnology, Haixia Institute of Science and Technology, Fujian Agriculture and Forestry University, Fuzhou 350002, China

Dear Editor,

Hemp (*Cannabis sativa* L.), a diploid dioecious species with a karyotype of 2*n* = 20, has garnered significant attention in genomics research due to its agricultural versatility and diverse secondary metabolites. Recent genome sequencing efforts have primarily focused on female plants and CBD-rich cultivars, leaving critical gaps in understanding male-specific genomic structures. Notably, prior assemblies lacked haplotype-resolved Y chromosome sequences, impeding investigations into sex determination mechanisms—a pivotal factor for breeding and pharmaceutical applications. Comparative genomic analyses reveal that male-specific regions (MSRs) in other species (e.g. 761 kb in *Populus euphratica* and 17.42 Mb in *Spinacia oleracea*) often harbor key sex-determining genes [1, 2]. Our study addresses this gap by presenting the complete, haplotype-resolved Y chromosome assembly for seed-type hemp. This breakthrough enables the identification of candidate sex-determination loci and provides a robust framework for dissecting gender-related traits in *Cannabis sativa*.

## Genome assembly and quality assessment of seed hemp ‘Yushe’

### Genomic data production and size estimation

**Figure 1:**
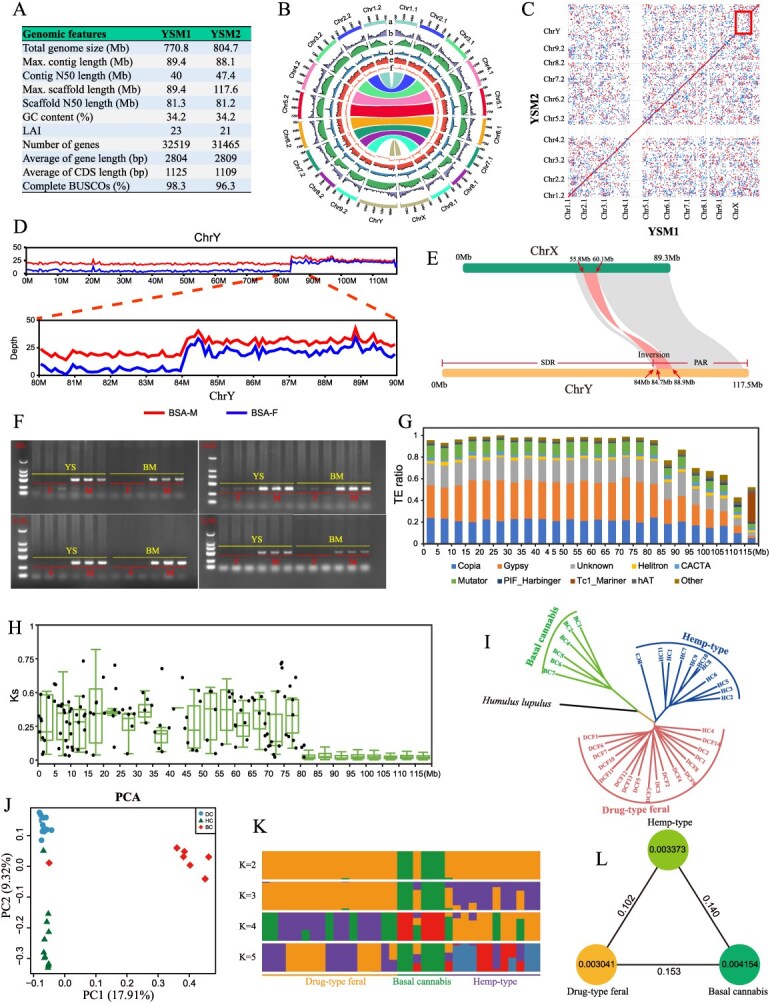
Genome assembly and population genomic analysis of the male plant in seed hemp. (A) *De novo* assembly of YSM1 and YSM2 with annotation of 32 519 and 31 465 protein-coding genes, respectively. (B) Haplotype-resolved genomic features. (C) Collinearity analysis of YSM1 and YSM2. (D) Differential depth of coverage across Y chromosome in the male genome using BSA data. (E) Synteny plot between the X and Y chromosomes, including an inversion of the homologous region. The divergence time of X/Y was 37.7 Mya (Ks = 0.46). (F) Molecular marker validation confirms: 84 Mb male-specific SDR, 100% concordance between ‘Yushe’ (YS) and ‘Bama’ (BM) cultivars. (G) Repeat types and repeat density in Y chromosome. X axis: Y chromosome; Y axis: TE ratio. (H) Ks of homolog gene pairs between X and Y. The x-axis has a window every 5 Mb. (I–L) Phylogenetic relationships and population structure in *Cannabis Sativa*: Phylogenetic analysis reveals distinct evolutionary trajectories among chemotype subgroups (I), while principal component analysis (PCA) demonstrates clear genomic separation between fiber-rich and psychoactive cultivars (J). Population structure inference identifies three genetically distinct clusters (*K* = 3) among 35 accessions, corroborating previous chemotaxonomic classifications. Nucleotide diversity and population divergence across the three groups. The value within the circle indicates the nucleotide diversity (π) of the population, and the value between the two circles indicates population divergence (L).

We generated 26.27 Gb (32× coverage) of high-fidelity (HiFi) PacBio Sequel II reads and 39.57 Gb (49× coverage) of ultra-long Oxford Nanopore Technology (ONT) reads from the Shanxi local male seed hemp variety ‘Yushe’ (Table S1). K-mer frequency analysis estimated the genome size at 816 Mb with 2.14% heterozygous sites (Fig. S1). Due to the observed high heterozygosity in the YSM population, we employed the hifiasm software (v0.16, default parameters) for *de novo* genome assembly and haplotype phasing, resulting in two distinct haplotypes: YSM1 (770 Mb) and YSM2 (804 Mb) (Fig. 1A and B, Fig. S2, Tables S2 and S3).

### Assembly quality metrics

Benchmarking Universal Single-Copy Orthologs (BUSCO) evaluation revealed that 98.8% and 95.8% of conserved BUSCO genes were complete in YSM1 and YSM2, respectively (Table S4). Notably, the long terminal repeat Assembly Index (LAI) values of YSM1 (23) and YSM2 (21) significantly exceeded those reported for previously published cannabis genomes (Table S5). Transcriptome mapping analyses confirmed high assembly quality, with 92.21% and 92.03% of seed hemp RNA-seq transcripts aligning to YSM1 and YSM2, respectively (Table S6). When contrasted with existing cannabis reference genomes, our YSM assemblies demonstrated substantial improvements across multiple quality metrics (Table S7).

## Comparative genomic analysis of YSM1 and YSM2 haplotypes of seed hemp

Through a multi-methodological approach integrating *de novo* prediction, homology-based searches, and RNA-Seq assembly validation, we systematically identified 32 519 and 31 465 protein-coding genes in the YSM1 and YSM2 haplotypes, respectively (Table S8). Structural characterization revealed mean intron lengths of 495.68 bp (YSM1) and 513.26 bp (YSM2), with corresponding average intron counts of 3.39 and 3.31 per gene (Table S6).

Functional annotation was successfully completed for 96.1% of predicted genes in each haplotype (31 233/32 519 for YSM1 and 30 235/31 465 for YSM2), achieved through rigorous alignment against multiple public databases (Table S8). The completeness of gene prediction was further validated by BUSCO analysis, which demonstrated 98.3% and 96.3% full-length sequence coverage for YSM1 and YSM2 assemblies, respectively (Table S9).

Notably, our analysis revealed substantial repetitive sequence content in both haplotypes: accounting for 574.53 Mb (74.53%) of the YSM1 assembly and 614.23 Mb (76.33%) of YSM2 (Table S10). Transposable elements constituted the predominant component of these repeats in both assemblies, consistent with patterns observed in other complex plant genomes.

## Identification and characterization of the sex-determining region (SDR) in seed hemp

Through comprehensive collinearity analysis of the YSM1 and YSM2 haplotype genomes, we observed high synteny across autosomes, while the X and Y chromosomes exhibited collinearity only at the pseudo-autosomal region (PAR) terminus (Fig. 1C). To precisely delineate the SDR, we conducted bulked segregant analysis (BSA) using pooled DNA from 50 female (BSA-F) and 50 male (BSA-M) seed hemp plants. When aligned to the YSM1 reference genome, chromosomal coverage remained uniform; however, using YSM2 as reference revealed markedly reduced coverage in the initial 84 Mb segment of the Y chromosome, with BSA-F reads showing significantly lower coverage than BSA-M reads in this region (Fig. 1D, Fig. S3). Validation through individual resequencing of 11 male and 11 female plants from diverse genetic backgrounds confirmed male-biased coverage depth in this 84 Mb region (Fig. S4). Homology-based collinearity analysis between X and Y chromosomes further highlighted the structural uniqueness of this Y-chromosomal segment (Fig. 1E). Male-specific amplification patterns from SDR-targeted random primer PCR assays provided additional evidence (Fig. 1F, Table S11).

This study identifies an 84 Mb region on the Y chromosome (designated as the SDR) in seed hemp, which constitutes 71% of the total Y chromosome length. Unlike previous studies that only approximated the SDR region, our research is the first to clearly define its boundaries. Our findings align with prior reports demonstrating extensive non-recombination between X and Y chromosomes in plants [3]. Through integrated analysis of comparative genomics and RNA-seq data, we identified a Y-linked gene, *CsaJGB* (*Csa.2MYG00468*), exhibiting male-specific expression patterns. Quantitative PCR validation confirmed significantly higher expression levels of *CsaJGB* in male floral tissues compared to female counterparts (*P* < 0.01, Welch’s *t*-test). These results strongly suggest that *CsaJGB* may play a pivotal role in sex determination mechanisms in hemp, either as a primary determinant or a key regulator in the sex differentiation pathway.

## Comparative genomics of sex-determining regions in seed hemp

### Structural characteristics of the SDR

The SDR on the Y chromosome in seed hemp exhibits exceptionally high repetitive element density (91.4%), with LTR/Gypsy retrotransposons constituting 34.77% of this repetitive landscape (Fig. 1G). Comparative annotation revealed 570 protein-coding genes in the Y-SDR versus 1529 in the X-SDR, representing a 63% gene loss from the Y chromosome since recombination cessation with the X [4]. Within the SDR, we identified 150 gametolog pairs, 19 Y-specific genes, and 34 X-specific genes. In contrast, the PAR contained 1897 gametolog pairs, 121 Y-specific genes, and 123 X-specific genes (Fig. S5, Table S12).

### Evolutionary divergence analysis

To estimate the evolutionary timeline of sex chromosome divergence, we calculated the synonymous substitution rate (Ks) for 150 gene pairs between SDR and X chromosome counterpart. After outlier removal, the mean Ks was 0.46 (Fig. 1H, Table S13). Using the 63.5 Mya divergence time between cannabis and mulberry as a calibration point, we determined that X–Y chromosomal separation in seed hemp occurred approximately 37.7 Mya (95% CI, 31.1–44.3 Mya) [5].

### Chromosomal rearrangements

A significant structural rearrangement was detected within the SDR, comprising two massive inverted segments (25.6 and 54.0 Mb) relative to the X chromosome (Table S14). This inversion event resulted in a change in the positions of 65 pairs of genes on the X and Y chromosomes, with the breakpoints located between positions 1-65 and 66-150 in the SDR. Notably, these inverted regions exhibit differential gene content, with the larger segment (54.0 Mb) containing 78% of the Y-specific genes identified in the SDR.

The observed high repetitive content (91.4%) and extensive gene loss (63%) in the Y-SDR align with theoretical models of sex chromosome evolution. The 25.6 Mb inversion segment shows enrichment in stress-responsive genes (*P* < 0.05, FDR-corrected), suggesting potential roles in sex-specific environmental adaptations.

## Genomic landscape of sex chromosomes in *Cannabis sativa*

Our resequencing analysis of 35 diverse cannabis accessions (Table S15) revealed distinct evolutionary patterns between sex chromosomes [6]. Alignment to the X chromosome assembly identified 1 635 026 high-confidence variants (18.3 variants/kb), comprising 1 422 211 SNPs, 98 894 insertions, and 113 921 deletions (collectively 212 815 InDels). In contrast, Y chromosome mapping detected 1 060 233 variants (9.0 variants/kb), including 923 502 SNPs with 63 454 insertions and 73 277 deletions (total 136 731 InDels).

Using the X and Y chromosomes as references, we estimated the average nucleotide diversity (π) to be 0.0034 and 0.0008, respectively. This indicates that the X chromosome has higher genetic diversity, which is consistent with theoretical predictions of sex chromosome evolution. This pattern likely reflects both: (i) Intensive artificial selection during cannabis domestication, particularly targeting male plants for fiber or drug traits, (ii) Progressive Y chromosome degeneration following suppression of recombination after sex chromosome differentiation (~37.7 Mya).

Phylogenetic reconstruction using X chromosomal markers (selected for higher π values) resolved three distinct subpopulations: basal cannabis (ancestral genotypes), industrial hemp-type (fiber cultivars), drug-type feral (psychoactive varieties). Notably, these groups showed comparable nucleotide diversity (Fig. 1I–L), suggesting parallel domestication pressures. Comparative genomics identified stronger selective constraints in the SDR versus PAR, evidenced by the higher π values in PAR and positive Tajima's D in SDR, indicating balancing selection.
